# Curcumin induces apoptosis in gallbladder carcinoma cell line GBC-SD cells

**DOI:** 10.1186/1475-2867-13-64

**Published:** 2013-06-26

**Authors:** Tian-Yu Liu, Zhu-Jun Tan, Lin Jiang, Jian-Feng Gu, Xiang-Song Wu, Yang Cao, Mao-Lan Li, Ke-Jin Wu, Ying-Bin Liu

**Affiliations:** 1 Laboratory of General Surgery and Department of General Surgery, Xinhua Hospital, Affiliated to Shanghai Jiao Tong University, School of Medicine, No. 1665 Kongjiang Road, Shanghai 200092, China; 2 Department of General Surgery, Changshu Hospital, Affiliated to Suzhou University, Changshu, China; 3 Research Institute of Biliary Tract Disease, Affiliated to Shanghai Jiao Tong University, School of Medicine, No. 1665 Kongjiang Road, Shanghai 200092, China

**Keywords:** Curcumin, Gallbladder carcinoma GBC-SD cell, Proliferation, Apoptosis

## Abstract

**Background:**

Gallbladder carcinoma is a malignant tumor with a very low 5-year survival rate because of the difficulty with its early diagnosis and the very poor prognosis of the advanced cancer state. The aims of this study were to determine whether curcumin could induce the apoptosis of a gallbladder carcinoma cell line, GBC-SD, and to clarify its related mechanism.

**Methods:**

First, the anti-proliferative activities of curcumin-treated and untreated GBC-SD cells were determined using the MTT and colony formation assays. Then, the early apoptosis of cells was detected by the annexin V/propidium iodide double-staining assay and Hoechst 33342 staining assay. Detection of mitochondrial membrane potential was used to validate the ability of curcumin on inducing apoptosis in GBC-SD cells. Cell cycle changes were detected by flow cytometric analysis. Finally, the expressions of the apoptosis-related proteins or genes caspase-3, PARP, Bcl-2, and Bax were analyzed by western blot and quantitative real time PCR assay. Statistical analyses were performed using the Student’s *t*-test for comparison of the results obtained from cells with or without curcumin treatment.

**Results:**

The MTT assay revealed that curcumin had induced a dose- and a time-dependent decrease in cell viability. Colony counting indicated that curcumin had induced a dose-dependent decrease in the colony formation ability in GBC-SD cells. Cells treated with curcumin were arrested at the S phase, according to the flow cytometric analysis. A significant induction of both the early and late phases of apoptosis was shown by the annexin V-FITC and PI staining. Morphological changes in apoptotic cells were also found by the Hoechst 33342 staining. After treatment with curcumin fluorescence shifted from red to green as ΔΨm decreased. Furthermore, western blot and quantitative real time PCR assays demonstrated that the curcumin induced apoptosis in GBC-SD cells by regulating the ratio of Bcl-2/Bax and activating the expression of cleaved caspase-3.

**Conclusions:**

Taken together, the results indicate that curcumin may be a potential agent for the treatment of gallbladder cancer.

## Background

Gallbladder carcinoma is one of the most common malignant tumors of the biliary system and is the fifth most common malignancy of the gastrointestinal tract [1,2]. Early gallbladder carcinoma is asymptomatic or manifests only as an abdominal discomfort. Some patients can develop the symptom of acute or chronic cholecystitis, which is easy to ignore or miss. In the later period, patients can develop abdominal pain, jaundice, and angular, but most of the patients have no surgical opportunities. The prognosis of advanced gallbladder carcinoma is very poor, [3-5] and the 5-year survival rate is only about 5% [6]. So far, surgical resection is the only treatment that offers a hope for cure [7]. Moreover, the majority of patients have frequent recurrences following surgery and unsatisfactory results following chemotherapy or radiotherapy [8]. Therefore, more research about the occurrence of gallbladder carcinoma and the mechanism of its development, as well as finding effective treatments and drugs, is an important need.

Curcumin, a phenolic compound present in Zingiberaceae *Curcuma longa*, rhizoma zedoariae, turmeric, etc., has been shown to have anticarcinogenic [9-11] and anti-inflammatory properties [12], including an inhibitory effect on the production of various cytokines. Curcumin has attracted much attention because of its low price and low toxicity, as well as its wide pharmacological and potential anticancer effects. It is believed that the anticancer mechanism of curcumin is mainly in inducing the apoptosis of cancer cells [13-15] and suppressing metastasis [16-18]. The apoptosis induced by curcumin is due to the activation of a multi-signal transduction pathway. Curcumin induces apoptosis in breast cancer cell lines, and the activation of apoptosis was confirmed by PARP-1 cleavage and by the increased ratio between the pro-apoptotic Bax and the anti-apoptotic Bcl-2 proteins [19]. Moreover, apigenin and curcumin synergistically induced cell death and apoptosis and also blocked cell cycle progression at the G2/M phase of A549 cells [20]. Although curcumin has been found to induce apoptosis in several types of cancers, the molecular apoptotic mechanisms of curcumin in the gallbladder carcinoma cell line GBC-SD have not previously been investigated.

The goals of this study were to determine whether curcumin could induce apoptosis in GBC-SD cells and to clarify the related mechanism, which may offer a promising new approach in the effective treatment of gallbladder carcinoma.

## Results

### Effect of curcumin on the viability of GBC-SD cells

**Figure 1:**
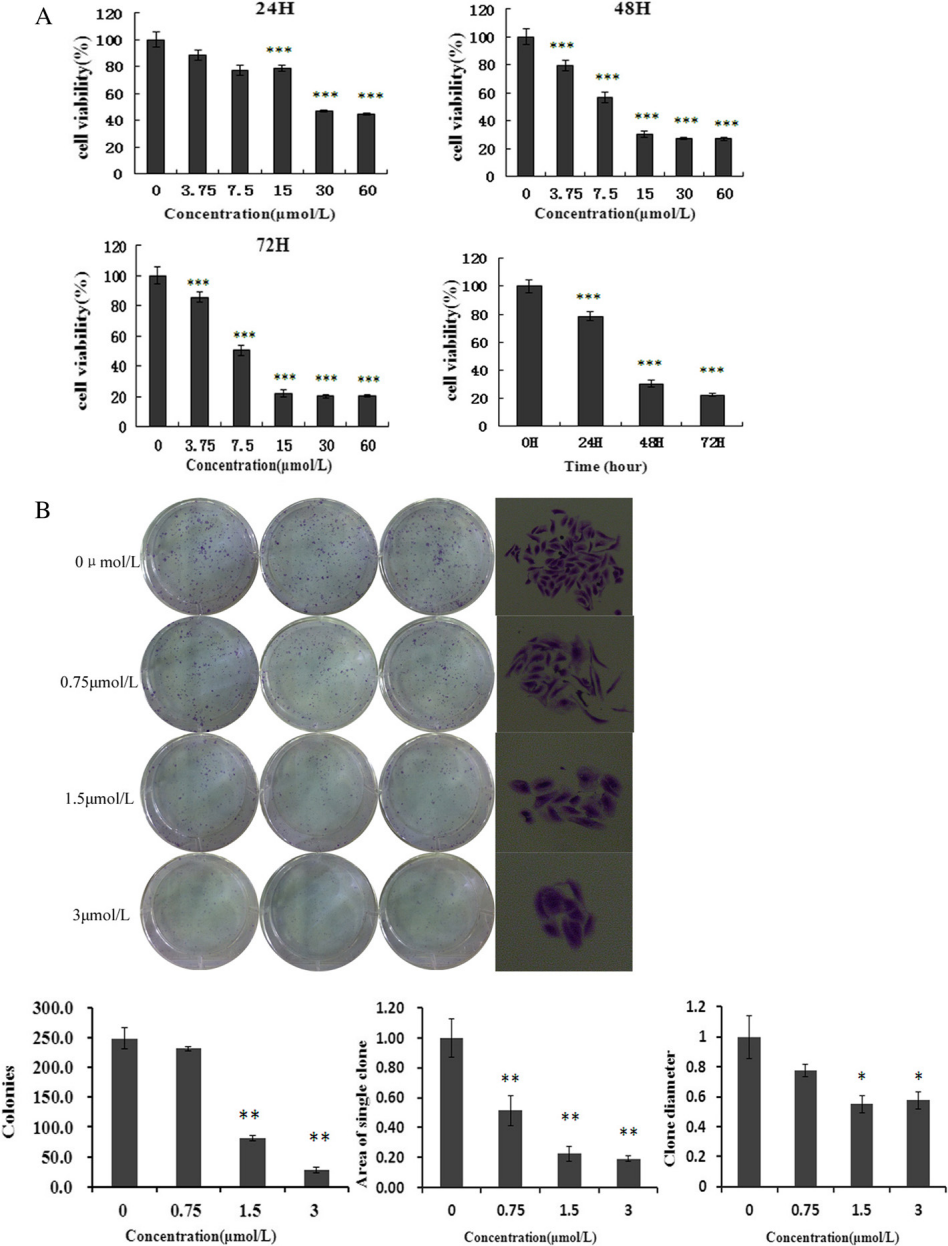
**Curcumin inhibits the proliferation of GBC-SD cells. (A)** Cells were treated with varying concentrations of curcumin, and the cell proliferation and IC_50_ were determined by MTT assay on days 1, 2, and 3. Each value represents the mean ± SD (n = 3). **(B)** Curcumin suppressed colony formation of GBC-SD cells. Cells were treated with curcumin (0.75, 1.5, and 3 μmol/L) and were allowed to form colonies in fresh medium for 14 days. The photomicrographic difference (Left panel) and influence of colonies (mean ± SD, n = 3) (Right panel) in colony formation are shown.

The effects of curcumin on the growth of human GBC-SD cells in vitro were tested. As shown in Figure 1(A), after treatment for 24, 48, and 72 h, curcumin induced a dose- and a time-dependent decrease in the viability of the GBC-SD cells, as analyzed by the MTT assay. As shown in Figure 1(B), the ability of GBC-SD cells to form colonies in the presence of curcumin was detected with the flat plate colony formation assay. The colony count indicated that curcumin had induced a dose-dependent decrease in the colony formation ability. Moreover, statistical analysis demonstrated that the mean sizes of the control colonies were larger than those of the curcumin-treated group. The findings support the fact that curcumin may exert a significant influence on GBC-SD cell proliferation.

### Effect of curcumin on cell cycle distribution in GBC-SD cells

**Figure 2:**
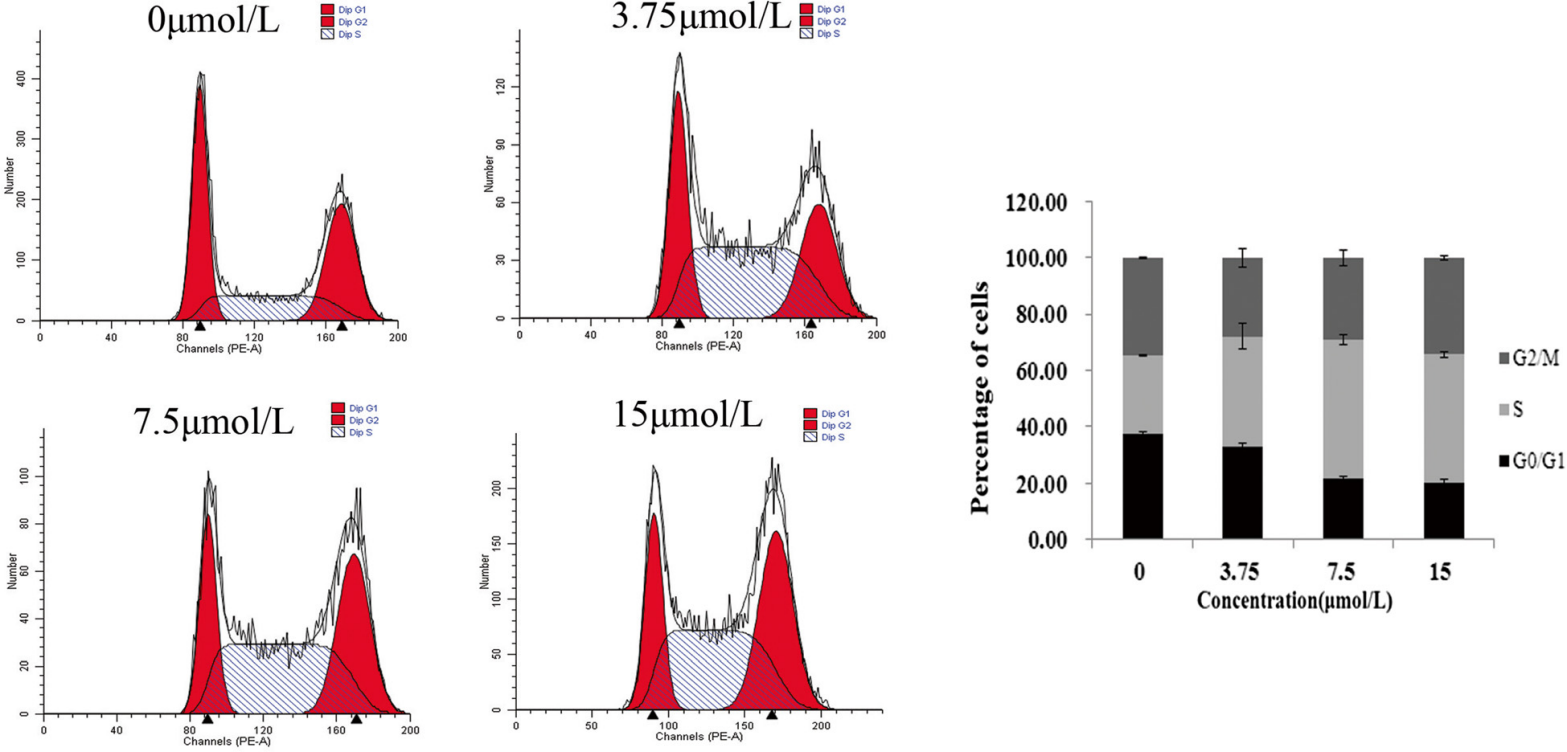
**Curcumin induces S-phase arrest in GBC-SD cells.** Cells were treated with , 3.75, 7.5 and 15 μmol/L curcumin for 48 h and the DNA content was analyzed by flow cytometry. The percentage of cells in the G1, S, and G2/M phases of the cell cycle are shown. These results were from 1 representative experiment of 3 independent trials.

To assess whether curcumin affects cell cycle progression, flow cytometric analysis was carried out. The results showed a significant decrease in the number of cells in the proliferative G0/G1 phase and a significant increase in the number of cells in the S phase, after 48 h of treatment with curcumin (Figure 2). These results indicate that curcumin arrests the cell cycle at the S phase.

### Effect of curcumin on apoptosis in GBC-SD cells

**Figure 3:**
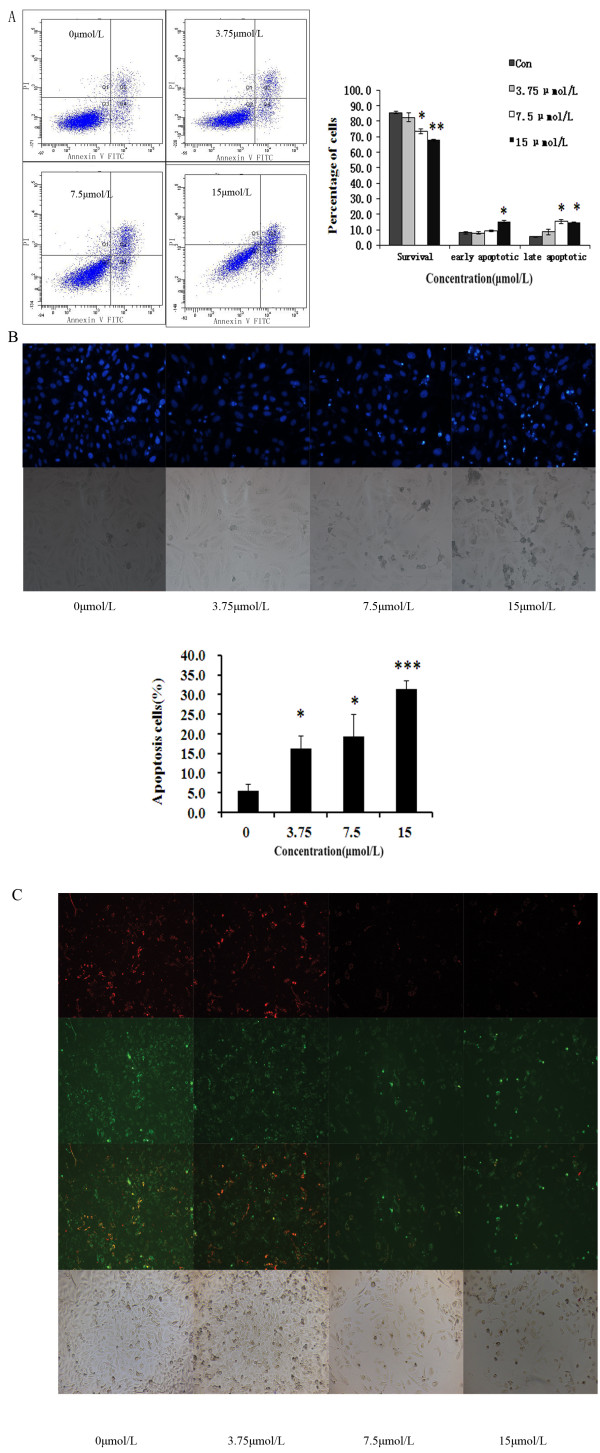
**Curcumin induces apoptosis in GBC-SD cells. (A)** Cells were incubated with curcumin (3.75, 7.5, and 15 μmol/L) for 48 h, followed by staining with annexin-V/PI. The Q3 quadrant (annexin V−/PI−), Q4 quadrant (annexin V+/PI−) and Q2 quadrant (annexin V+/PI+) indicate the percentage of normal cells, early apoptosis and late apoptosis, respectively. **(B)** Apoptotic nuclear morphology changes induced by curcumin (3.75, 7.5, and 15 μmol/L) treatment for 48 h, were observed by Hoechst 33342 staining in GBC-SD cell lines. **(C)** Analysis of the mitochondrial membrane potential (ΔΨm). GBC-SD cells were treated with curcumin (3.75, 7.5, and 15 μmol/L) for 48h and then stained with JC-1. Red fluorescence represents mitochondria with intact membrane potential. Green fluorescence represents de-energized mitochondria. Images were taken with a fluorescence microscope.

To confirm these results, we evaluated the effects of curcumin on apoptosis in GBC-SD cells by using annexin V-FITC and propidium iodide staining. Normal live cells, phosphatidyl serine (PS) is located on the cytoplasmic surface of the cell membrane. However, in apoptotic cells, PS is translocated from the inner to the outer leaflet of the plasma membrane, thus exposing PS to the external cellular environment. The human anticoagulant, annexin-V, is a 35–36 kDa Ca2+−dependent phospholipid binding protein that has a high affinity for PS. Annexin-V labeled with a fluorophore or biotin can identify apoptotic cells by binding to PS exposed on the outer leaflet.In addition, the red-fluorescent propidium iodide (PI) nucleic acid binding dye is impermeant to live cells and apoptotic cells, but stains dead cells with red fluorescence, binding tightly to the nucleic acids in the cell. After staining a cell population with annexin V and PI, apoptotic cells show green fluorescence, dead cells show red and green fluorescence, and live cells show little or no fluorescence. These populations can easily be distinguished using a flow cytometer. In the scatter plot of double variable flow cytometry, Q3 quadrant(FITC - / PI -) shows living cells; Q2 quadrant (FITC + /PI + ) stands for late apoptotic cells; and Q4 quadrant(FITC + /PI -) represents early apoptotic cells. As assessed by flow cytometry and shown in Figure 3(A), a marked dose-dependent increase in both the early and late stages of apoptosis was obvious in GBC-SD cells after curcumin treatment compared with control cells.

Morphological changes in the apoptotic cells were revealed by the Hoechst 33342 staining, as shown in Figure 3(B). In the untreated GBC-SD cells, the nuclei were stained weak homogeneous blue, whereas in the group treated with curcumin, bright chromatin condensation and nuclear fragmentation were observed, the rates of which increased in a dose-dependent manner.

### Effects of curcumin on mitochondrial membrane potential(ΔΨm)

To validate the ability of curcumin on inducing apoptosis in GBC-SD cells, we performed a cellular functional assay. JC-1 probe is a fluorescent cationic dye that can selectively accumulate into mitochondria by electrochemical gradient and changes color from red to green as ΔΨm decreases. As illustrated in Figure 3(C), untreated GBC-SD cells exhibited red fluorescence. After treatment with.

### Effect of curcumin on the signal pathway of caspase and Bcl-2 family members in GBC-SD cells

**Figure 4:**
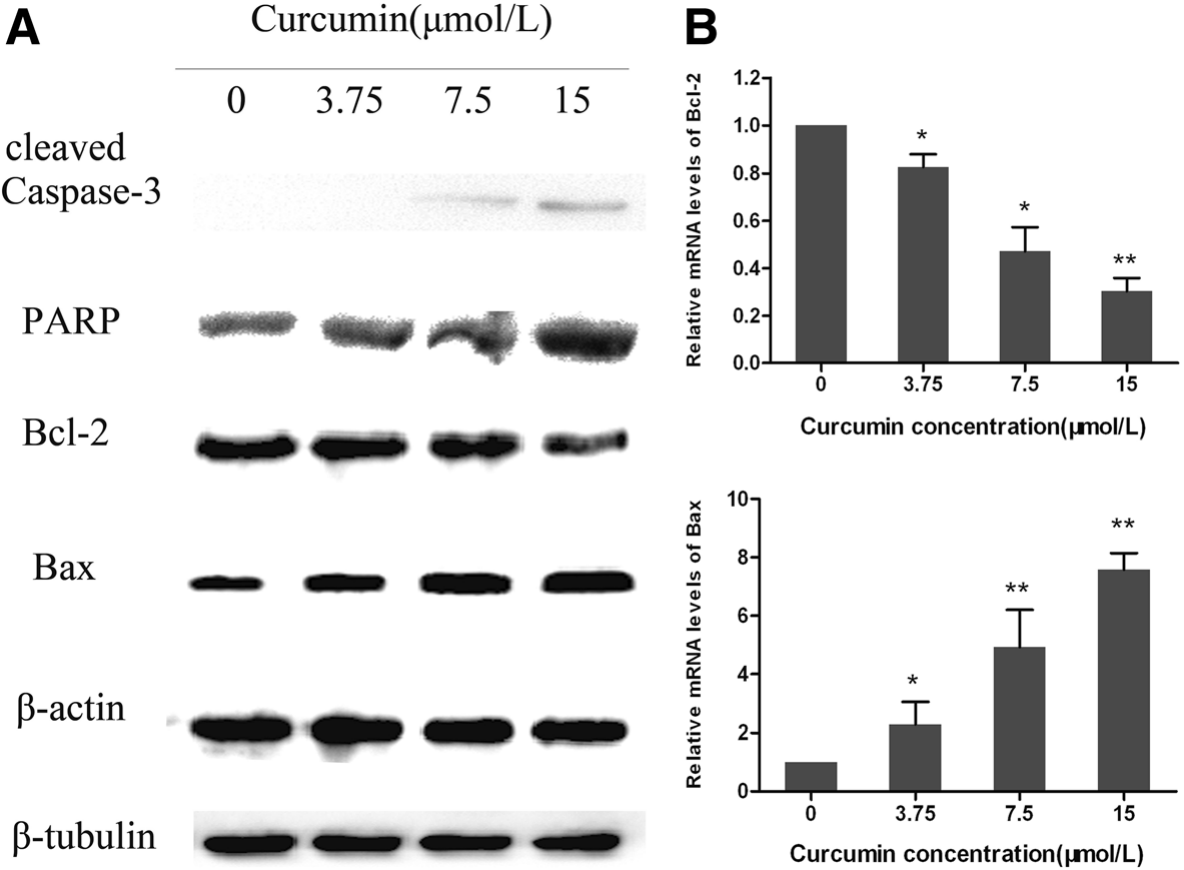
**Curcumin induces the activation of apoptosis-related proteins and mRNA in GBC-SD cells. (A)** After treatment with curcumin (0, 3.75, 7.5, and 15 μmol/L) for 48h, cell lysates were prepared and western blot analysis was performed against Bcl-2, Bax, caspase-3, and PARP. β-Actin and β-tubulin were used as a loading control. **(B)** Quantitative real-time PCR was used to examine the expression of Bax and Bcl-2 mRNA. Curcumin upregulated mRNA expression of Bax, and downregulated mRNA expression of Bcl-2.

To investigate the possible mechanism of curcumin’s apoptotic effect on the GBC-SD cells, the expressions of apoptosis-related proteins (viz., PARP, caspase-3, Bax, and Bcl-2) were assessed by western blot analysis. As illustrated in Figure 4(A), treatment with curcumin resulted in a downregulation of Bcl-2, and an upregulation of Bax, cleaved caspase-3, and PARP, which may be partially responsible for the apoptotic tendency of the GBC-SD cells. Real-time quantitative PCR was used to quantify the levels of Bax and Bcl-2 mRNA. Figure 4(B) showed that the expression of Bax mRNA was upregulated in the curcumin treatment group while Bcl-2 mRNA was downregulated.

## Discussion

To the best of our knowledge, this is the first study to reveal the ability of the natural compound curcumin to induce apoptosis in GBC-SD cells. In recent years, studies have shown that curcumin can inhibit the growth of a variety of tumor cells [9,21,22], as well as induce cell differentiation[23] and apoptosis in some tumors [24-27]. Curcumin can also be used as a natural antitumor drug [28]. Here, we have shown the biochemical and molecular mechanisms of apoptosis induction by curcumin in GBC-SD cells.

Curcumin manifests its antitumor effect through the induction of cell apoptosis in vitro. Our experiment showed a significant inhibition of cell proliferation in a dose- and time-dependent manner, thereby suggesting that treatment with curcumin inhibited the growth and reduced the viability of GBC-SD cells. The cell apoptosis experiment showed that curcumin had the biological activity of inducing tumor cell apoptosis. We used flow cytometry to detect the cell cycle, and the results showed a significant decrease in the number of cells in the proliferative G0/G1 phase and a significant increase in the number of cells in the S phase, after 48 h of treatment with curcumin. The result suggested that curcumin could block cells in the S phase, which prevented DNA from replicating properly, thus inhibiting tumor growth.

Cell apoptosis is an autonomous cell death process, which can be induced by a variety of drugs and physical and chemical factors. The induction of apoptosis has been described as a standard and best strategy in anticancer therapy [29,30]. The family of cysteine-containing aspartate-specific proteases (caspase) contains many members that are closely related with cell apoptosis [31,32]. So far, 10 members have been identified in humans. Caspase-3, a protein on the common path of cell apoptosis, is one of the most important members and the key executor of cell apoptosis. Caspase-3 usually exists in the cytoplasm in the form of an inactive zymogen. When activated by the many external apoptosis signals, caspase-3 can induce the inactivation of many key proteases in the cytoplasm, cell nucleus, and cytoskeleton, and finally cause the apoptosis of cells. In our study, the results showed that the change of caspase-3 expression was in accordance with the tendency of changes in cell apoptosis. The cleavage of PARP was increased accordingly. The Bcl-2 gene family is one of the best studied of the anti-apoptosis genes, and according to the members’ different biological effects, it is divided mainly into the Bax, Bcl-2, and Bid proteins. Among them, the apoptosis-promoting protein Bax and the anti-apoptotic protein Bcl-2 play an important role in regulating cell apoptosis [33,34]. The occurrence and severity of apoptosis depends on the ratio of Bcl-2/Bax [35]. When this ratio is reduced, the caspase apoptosis proteins can be activated. Through the western blot assay, it was found that the expression of Bcl-2 was reduced and that of Bax was promoted in GBC-SD cells treated with curcumin, thus decreasing the Bcl-2/Bax ratio significantly.

## Conclusion

In conclusion, this study suggests that curcumin inhibits the proliferation of GBC-SD cells and arrests the cell cycle in the S phase. Curcumin induces GBC-SD cell apoptosis mainly by activating the Bcl-2 and caspase-3 pathways. Other apoptotic mechanisms remain to be researched further.

## Methods

### Cell lines and culture

GBC-SD cells were purchased from the Shanghai Cell Institute Country Cell Bank. The cells were cultured in high-glucose DMEM (Gibco, USA) supplemented with 10% fetal bovine serum (Gibco, USA), 100 μg/mL streptomycin and 100 U/mL penicillin (Hyclone, USA), at 37°C, under a 5.0% CO_2_ atmosphere.

### Drugs and antibodies

**Figure 5:**
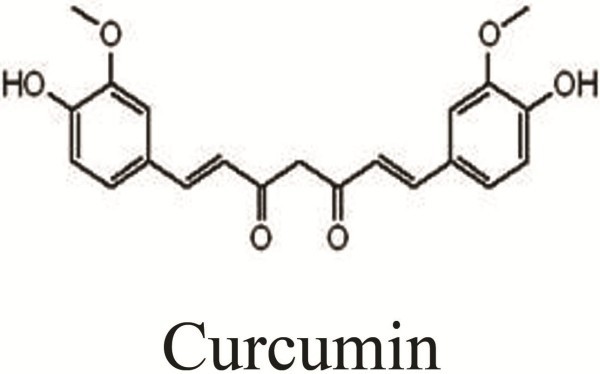
**Chemical structure of curcumin.** The molecular formula of curcumin is C_21_H_20_O_6_ and its molecular weight is 368.37.

The curcumin was purchased from Sigma-Aldrich (St. Louis, USA), dissolved in DMSO as a stock concentration of 100 mmol/L, and stored in the dark at −20°C. Figure 5 shows the chemical structural of curcumin. The final curcumin concentrations used for the different experiments were prepared by diluting the stock solution with high-glucose DMEM. The antibodies used for western blotting were as follows: rabbit anti-caspase-3, anti-Bcl-2, anti-Bax, anti-PARP, anti-tubulin and mouse anti-β-actin. All the antibodies were purchased from Cell Signaling Technology.

### 3-(4,5-Dimethylthiazol-2-yl)-2,5-diphenyltetrazolium (MTT) assay

Drug sensitivity was determined using the MTT assay [36]. Briefly, cells were trypsinized and plated into 96-well plates (Corning, USA) at a density of 5 × 10^3^ cells per well. The cells were cultured overnight and then replenished with fresh medium containing various concentrations (0, 3.75, 7.5, 15, 30, and 60 μmol/L) of curcumin for 24, 48, and 72 h. Thereafter, 20 μL of MTT (Sigma-Aldrich) dissolved in PBS at 5 mg/mL was added directly to all the wells, and the plates were incubated for 4 h at 37°C. The formazan crystals that formed were dissolved in 100 μL of DMSO after removal of the supernatant. The optical density was recorded at 490 nm on a microplate reader (Bio-Tek, USA). The results represent the average of 3 independent experiments done over multiple days. The percentage of cell viability was calculated as follows:

$$\mathrm{cell}\;\mathrm{viability}\;\left(\%\right)=\frac{\mathrm{OD}\;\mathrm{of}\;\mathrm{treatment}}{\mathrm{OD}\;\mathrm{of}\;\mathrm{control}}\times 100$$

### Colony formation assay

Cells in the logarithmic growth phase were digested into a single-cell suspension with a trypsin-EDTA (Gibco, USA) solution, and then 2 mL of the cell suspension was seeded onto 6-well plates (Corning, USA) at a density of 200 cells/mL. After adherence, the cells were treated with curcumin (0.75, 1.5, and 3 μmol/L) for 48 h and then cultured for 15 days. Thereafter, the cells were fixed with 10% formalin and stained with 0.1% crystal violet (Sigma-Aldrich). After washing, the plates were air dried, and digital images were taken of stained single clones observed under a microscope (Leica, Germany). The results represent the average of 3 independent experiments done over multiple days.

### Cell cycle analysis

GBC-SD cells were treated with curcumin (3.75, 7.5, and 15 μmol/L) for 48 h. Then, the cells were collected and fixed with cold 70% ethanol and stored at −20°C. The cells were then washed and resuspended in cold PBS and incubated at 37°C for 30 min with 10 mg/mL RNase and 1 mg/mL propidium iodide (Sigma-Aldrich). DNA content analysis was performed by flow cytometry (BD, San Diego, USA). The percentage of cells in the different cell cycle phases was determined using the Cell Quest acquisition software (BD Biosciences).

### Flow cytometric analysis of cell apoptosis

The annexin V/propidium iodide assay was performed according to the manufacturer’s recommendation (Invitrogen, USA). Briefly, GBC-SD cells were plated into 6-well plates (Corning, USA) and incubated for 48 h with curcumin (3.75, 7.5, and 15 μmol/L). In brief, the cells were collected and then were washed with cold PBS, centrifuged, resuspended in 100 ul of binding buffer containing 2.5 ul FITC conjugated annexin-v and 1ul 100 ug/ml PI and incubated for 15 mins at room temperature in the dark. A total of at least 10 000 events were collected and analyzed by flow cytometry (BD, San Diego, USA).

### Detection of morphological apoptosis with Hoechst 33342 staining

After treatment with curcumin (3.75, 7.5, and 15 μmol/L) for 48 h, the GBC-SD cells were washed with PBS and fixed with methanol:acetic acid (3:1) for 15 min at room temperature. Fixed cells were washed with PBS and stained with 5 μg/mL of Hoechst 33342 stain for 10 min. Changes in the nuclei of cells after staining with Hoechst 33342 were observed using a fluorescence microscope (Leica, Germany).

### Detection of mitochondrial membrane potential(ΔΨm) variation with fluorescence microscopy

The ΔΨm was analyzed by fluorescence microscopy using the 5,50,6,60-tetrachloro-1,10,3,30-tetraethylbenzimidazolcarbocyanine iodide (JC-1) probe. After treatment with curcumin (3.75, 7.5, and 15 μmol/L) for 48 h, 5ul of the JC-1 staining solution(Beyotime,China) per ml culture of medium was added to each well and samples were then incubated in 5% CO2 incubator at 37°C for 20 min protected from light. After washing twice with buffer solution, GBC-SD cells were analyzed by using a fluorescence microscope (Leica, Germany).

### Reverse transcription and quantitative real-time polymerase chain reaction (qPCR)

Quantitative PCR was performed as previously described [37] qPCR was used to quantify the expression of BCL2 and Bax mRNA in the experimental groups. GBC-SD cells were treated with curcumin (3.75, 7.5, and 15 μmol/L) for 48 h. Total RNA was isolated using the RNA easy kit (Invitrogen, USA). First strand cDNA was synthesized from 500 ng total RNA using a PrimeScript® Reverse Transcriptase (TaKaRa, Japan). Quantitative real-time PCR was performed in a reaction volume of 20 μl including 2 μl cDNA. The primer sequences were as follows: BCL2 (forward-5’-CAA ATG CTG GAC TGA AAA ATT GTA-3’ , reverse-5’-TAT TTT CTA AGG ACG GCA TGA TCT-3’), BAX (forward-5’-GAC ACC TGA GCT GAC CTT GG-3’; reverse-5’-AGG AAG TCC AGT GTC CAG C-3’) and GAPDH (forward-5’-AAG CTC ATT TCC TGG TAT GACA-3’ , reverse-5’-TCT TAC TCC TTG GAG GCC ATGT-3’). PCR conditions were as follows: 95°C for 30sec followed by 40 cycles at 95°C for 5 sec, 60°C for 34 sec. Glyceraldehyde-3-phosphate dehydrogenase (GAPDH) was used as an internal reference gene to normalize the expression of apoptotic genes. Relative quantification of apoptosis-related genes was analyzed by the comparative threshold cycle (Ct) method. For each sample, the Ct value of the apoptotic gene was normalized using the formula: ΔCt = Ct (apoptotic genes) - Ct (GAPDH). To determine relative expression levels, the following formula was used: ΔΔCt = ΔCt (treated) - ΔCt (control). The value was used to plot the expression of apoptotic genes using the formula 2−ΔΔCt.

### Western blot analysis

Western blot was performed as previously described [38]. Breafly, GBC-SD cells were treated with various concentrations of curcumin (3.75, 7.5, and 15 μmol/L) for 48 h and then lysed in a sample buffer, followed by denaturation. The total protein concentration of the cell extracts was determined using the bicinchoninic acid assay system (Beyotime, China) with BSA as a standard. Equal quantities (80 μg protein per lane) of total proteins were separated by SDS-PAGE (8%, 12% gels) under reducing conditions. The proteins were then electrophoretically transferred to nitrocellulose membranes. The membranes were blocked with 5% skimmed milk, and incubated with anti-caspase-3, anti-Bcl-2, anti-Bax, anti-PARP, anti-β-actin and anti-β-tubulin antibodies, respectively (1:1000; Cell Signaling Technology) at 4°C overnight. This was followed by an incubation with goat anti-rabbit/anti-mouse secondary antibody conjugated with horseradish peroxidase (1:5000; Abcam). An equal loading of each lane was evaluated by immunoblotting the same membranes with β-actin antibodies after the detachment of previous primary antibodies. Photographs were taken and the optical densities of the bands were scanned and quantified with the Gel Doc 2000 (BioRad, USA).

### Statistical analysis

All values are expressed as the mean ± SD and they were analyzed by the Student’s *t-*test using SPSS version 13.0 software. A *p*-value of less than 0.05 was considered significant.

## Competing interests

The authors declare that they have no competing interests.

## Authors’ contributions

TZJ and LTY were responsible for the design of the experiments. LTY contributed to the execution of experiments, data statistics, and writing of the manuscript. JL participated in performing the experiment, and in the mapping and submission of the manuscript. LYB and TZJ were responsible for the funding application, and supervision and management of the project. All authors have contributed to and approved the final manuscript.

## References

[B1] Kaza RK Gulati M Wig JD Chawla YK Evaluation of gall bladder carcinoma with dynamic magnetic resonance imaging and magnetic resonance cholangiopancreatography Australas Radiol 2006 50 3 212 217 10.1111/j.1440-1673.2006.01564.x 16732816

[B2] Tan Z Li M Wu W Zhang L Ding Q Wu X Mu J Liu Y NLK is a key regulator of proliferation and migration in gallbladder carcinoma cells Mol Cell Biochem 2012 369 1–2 27 33 22733362 10.1007/s11010-012-1365-0

[B3] Wang JW Peng SY Li JT Wang Y Zhang ZP Cheng Y Cheng DQ Weng WH Wu XS Fei XZ Identification of metastasis-associated proteins involved in gallbladder carcinoma metastasis by proteomic analysis and functional exploration of chloride intracellular channel 1 Cancer Lett 2009 281 1 71 81 10.1016/j.canlet.2009.02.020 19299076

[B4] Wang JD Shi WB Shen J Zhuang PY Quan ZW Wang XF Zhou XP Li SG Liu YB Yang Y Evaluation of two modified ECF regimens in the treatment of advanced gallbladder cancer Med Oncol 2011 28 Suppl 1 S295 300 21136212 10.1007/s12032-010-9758-y

[B5] Dong P He XW Gu J Wu WG Li ML Yang JH Zhang L Ding QC Lu JH Mu JS Vimentin significantly promoted gallbladder carcinoma metastasis Chin Med J (Engl) 2011 124 24 4236 4244 22340393

[B6] Miller G Jarnagin WR Gallbladder carcinoma Eur J Surg Oncol 2008 34 3 306 312 10.1016/j.ejso.2007.07.206 17964753

[B7] Shimada K Nara S Esaki M Sakamoto Y Kosuge T Hiraoka N Extended right hemihepatectomy for gallbladder carcinoma involving the hepatic hilum Br J Surg 2011 98 1 117 123 10.1002/bjs.7262 21136566

[B8] Malka D Boige V Dromain C Debaere T Pocard M Ducreux M Biliary tract neoplasms: update 2003 Curr Opin Oncol 2004 16 4 364 371 10.1097/01.cco.0000129679.49651.50 15187892

[B9] Ye F Zhang GH Guan BX Xu XC Suppression of esophageal cancer cell growth using curcumin, (-)-epigallocatechin-3-gallate and lovastatin World J Gastroenterol 2012 18 2 126 135 10.3748/wjg.v18.i2.126 22253518 PMC3257439

[B10] Saha A Kuzuhara T Echigo N Fujii A Suganuma M Fujiki H Apoptosis of human lung cancer cells by curcumin mediated through up-regulation of "growth arrest and DNA damage inducible genes 45 and 153" Biol Pharm Bull 2010 33 8 1291 1299 10.1248/bpb.33.1291 20686221

[B11] Kumaravel M Sankar P Latha P Benson CS Rukkumani R Antiproliferative effects of an analog of curcumin in Hep-2 cells: a comparative study with curcumin Nat Prod Commun 2013 8 2 183 186 23513724

[B12] Guimaraes MR Coimbra LS de Aquino SG Spolidorio LC Kirkwood KL Rossa C Jr Potent anti-inflammatory effects of systemically administered curcumin modulate periodontal disease in vivo J Periodontal Res 2011 46 2 269 279 10.1111/j.1600-0765.2010.01342.x 21306385 PMC3086370

[B13] Debnath S Saloum D Dolai S Sun C Averick S Raja K Fata JE Dendrimer-curcumin 2013 A Water Soluble and Effective Cytotoxic Agent against Breast Cancer Cell Lines. Anticancer Agents Med Chem: Conjugate 10.2174/18715206113139990139 23387971

[B14] Faiao-Flores F Suarez JA Maria-Engler SS Soto-Cerrato V Perez-Tomas R Maria DA The curcumin analog DM-1 induces apoptotic cell death in melanoma Tumour Biol 2013 34 2 1119 1129 10.1007/s13277-013-0653-y 23359272

[B15] Wang WZ Li L Liu MY Jin XB Mao JW Pu QH Meng MJ Chen XG Zhu JY Curcumin induces FasL-related apoptosis through p38 activation in human hepatocellular carcinoma Huh7 cells Life Sci 2013 92 6–7 352 358 23352975 10.1016/j.lfs.2013.01.013

[B16] Zhang CY Zhang L Yu HX Bao JD Lu RR Curcumin inhibits the metastasis of K1 papillary thyroid cancer cells via modulating E-cadherin and matrix metalloproteinase-9 expression Biotechnol Lett 2013 35 7 995 1000 10.1007/s10529-013-1173-y 23474829

[B17] Cheng TS Chen WC Lin YY Tsai CH Liao CI Shyu HY Ko CJ Tzeng SF Huang CY Yang PC Curcumin-targeting pericellular serine protease matriptase role in suppression of prostate cancer cell invasion, tumor growth, and metastasis Cancer Prev Res (Phila) 2013 6 5 495 505 10.1158/1940-6207.CAPR-12-0293-T 23466486

[B18] Liu L Sun L Wu Q Guo W Li L Chen Y Li Y Gong C Qian Z Wei Y Curcumin loaded polymeric micelles inhibit breast tumor growth and spontaneous pulmonary metastasis Int J Pharm 2013 443 1–2 175 182 23287774 10.1016/j.ijpharm.2012.12.032

[B19] Masuelli L Benvenuto M Fantini M Marzocchella L Sacchetti P Di Stefano E Tresoldi I Izzi V Bernardini R Palumbo C Curcumin induces apoptosis in breast cancer cell lines and delays the growth of mammary tumors in neu transgenic mice J Biol Regul Homeost Agents 2013 27 1 105 119 23489691

[B20] Choudhury D Ganguli A Dastidar DG Acharya BR Das A Chakrabarti G Apigenin shows synergistic anticancer activity with curcumin by binding at different sites of tubulin Biochimie 2013 95 6 1297 1309 10.1016/j.biochi.2013.02.010 23485682

[B21] Subramaniam D Ponnurangam S Ramamoorthy P Standing D Battafarano RJ Anant S Sharma P Curcumin induces cell death in esophageal cancer cells through modulating Notch signaling PLoS One 2012 7 2 e30590 10.1371/journal.pone.0030590 22363450 PMC3281833

[B22] Han X Xu B Beevers CS Odaka Y Chen L Liu L Luo Y Zhou H Chen W Shen T Curcumin inhibits protein phosphatases 2A and 5, leading to activation of mitogen-activated protein kinases and death in tumor cells Carcinogenesis 2012 33 4 868 875 10.1093/carcin/bgs029 22298641 PMC3324444

[B23] Tu SP Jin H Shi JD Zhu LM Suo Y Lu G Liu A Wang TC Yang CS Curcumin induces the differentiation of myeloid-derived suppressor cells and inhibits their interaction with cancer cells and related tumor growth Cancer Prev Res (Phila) 2012 5 2 205 215 10.1158/1940-6207.CAPR-11-0247 22030090 PMC3273601

[B24] Quitschke WW Curcuminoid binding to embryonal carcinoma cells: reductive metabolism, induction of apoptosis, senescence, and inhibition of cell proliferation PLoS One 2012 7 6 e39568 10.1371/journal.pone.0039568 22768090 PMC3383725

[B25] Sahu RP Batra S Srivastava SK Activation of ATM/Chk1 by curcumin causes cell cycle arrest and apoptosis in human pancreatic cancer cells Br J Cancer 2009 100 9 1425 1433 10.1038/sj.bjc.6605039 19401701 PMC2694438

[B26] Miller M Chen S Woodliff J Kansra S Curcumin (diferuloylmethane) inhibits cell proliferation, induces apoptosis, and decreases hormone levels and secretion in pituitary tumor cells Endocrinology 2008 149 8 4158 4167 10.1210/en.2007-1760 18450960 PMC2488238

[B27] Milacic V Banerjee S Landis-Piwowar KR Sarkar FH Majumdar AP Dou QP Curcumin inhibits the proteasome activity in human colon cancer cells in vitro and in vivo Cancer Res 2008 68 18 7283 7292 10.1158/0008-5472.CAN-07-6246 18794115 PMC2556983

[B28] Bao B Ali S Banerjee S Wang Z Logna F Azmi AS Kong D Ahmad A Li Y Padhye S Curcumin analogue CDF inhibits pancreatic tumor growth by switching on suppressor microRNAs and attenuating EZH2 expression Cancer Res 2012 72 1 335 345 10.1158/0008-5472.CAN-11-2182 22108826 PMC3792589

[B29] Kelly PN Strasser A The role of Bcl-2 and its pro-survival relatives in tumourigenesis and cancer therapy Cell Death Differ 2011 18 9 1414 1424 10.1038/cdd.2011.17 21415859 PMC3149740

[B30] Strasser A Cory S Adams JM Deciphering the rules of programmed cell death to improve therapy of cancer and other diseases EMBO J 2011 30 18 3667 3683 10.1038/emboj.2011.307 21863020 PMC3173800

[B31] Kroemer G Galluzzi L Brenner C Mitochondrial membrane permeabilization in cell death Physiol Rev 2007 87 1 99 163 10.1152/physrev.00013.2006 17237344

[B32] Galluzzi L Vitale I Abrams JM Alnemri ES Baehrecke EH Blagosklonny MV Dawson TM Dawson VL El-Deiry WS Fulda S Molecular definitions of cell death subroutines: recommendations of the Nomenclature Committee on Cell Death 2012 Cell Death Differ 2012 19 1 107 120 10.1038/cdd.2011.96 21760595 PMC3252826

[B33] Korsmeyer SJ Shutter JR Veis DJ Merry DE Oltvai ZN Bcl-2/Bax: a rheostat that regulates an anti-oxidant pathway and cell death Semin Cancer Biol 1993 4 6 327 332 8142617

[B34] Lindsay J Esposti MD Gilmore AP Bcl-2 proteins and mitochondria–specificity in membrane targeting for death Biochim Biophys Acta 2011 1813 4 532 539 10.1016/j.bbamcr.2010.10.017 21056595

[B35] Walensky LD BCL-2 in the crosshairs: tipping the balance of life and death Cell Death Differ 2006 13 8 1339 1350 10.1038/sj.cdd.4401992 16763614

[B36] Plumb JA Cell sensitivity assays: the MTT assay Methods Mol Med 2004 88 165 169 14634227 10.1385/1-59259-406-9:165

[B37] Dong P Zhang Y Gu J Wu W Li M Yang J Zhang L Lu J Mu J Chen L Wogonin, an active ingredient of Chinese herb medicine Scutellaria baicalensis, inhibits the mobility and invasion of human gallbladder carcinoma GBC-SD cells by inducing the expression of maspin J Ethnopharmacol 2011 137 3 1373 1380 10.1016/j.jep.2011.08.005 21855619

[B38] Quan Z Gu J Dong P Lu J Wu X Wu W Fei X Li S Wang Y Wang J Reactive oxygen species-mediated endoplasmic reticulum stress and mitochondrial dysfunction contribute to cirsimaritin-induced apoptosis in human gallbladder carcinoma GBC-SD cells Cancer Lett 2010 295 2 252 259 10.1016/j.canlet.2010.03.008 20359814

